# Molecular Cloning and Expression of Three Polygalacturonase cDNAs from the Tarnished Plant Bug, *Lygus lineolaris*

**DOI:** 10.1673/031.008.2701

**Published:** 2008-04-01

**Authors:** Margaret L. Allen, Jeffrey A. Mertens

**Affiliations:** ^1^ Biological Control of Pests Research Unit, USDA-ARS, Stoneville, MS 38776 USA; ^2^ Fermentation Biotechnology Research Unit, USDA-ARS, Peoria, IL 61604

**Keywords:** pectin degrading enzyme, extraoral digestion, plant-insect interaction, saliva, semiquantitative PCR

## Abstract

Three unique cDNAs encoding putative polygalacturonase enzymes were isolated from the tarnished plant bug, *Lygus lineolaris* (Palisot de Beauvois) (Hemiptera: Miridae). The three nucleotide sequences were dissimilar to one another, but the deduced amino acid sequences were similar to each other and to other polygalacturonases from insects, fungi, plants, and bacteria. Four conserved segments characteristic of polygalacturonases were present, but with some notable semiconservative substitutions. Two of four expected disulfide bridge—forming cysteine pairs were present. All three inferred protein translations included predicted signal sequences of 17 to 20 amino acids. Amplification of genomic DNA identified an intron in one of the genes, *Llpg1*, in the 5′ untranslated region. Semiquantitative RT-PCR revealed expression in all stages of the insect except the eggs. Expression in adults, male and female, was highly variable, indicating a family of highly inducible and diverse enzymes adapted to the generalist polyphagous nature of this important pest.

## Introduction

Phytophagous insects derive nutritional resources by consuming and digesting plant compounds, some of which are complex, such as those found in cell walls. Pectin is a complex carbohydrate and a major component of the plant cell wall. Enzymes that degrade pectin form a large group that includes polygalacturonases (EC 3.2.1.15) that hydrolyze α(1–4) linked D-galacturonic acid units. Although polygalacturonases and other plant cell wall degrading enzymes have been identified and purified from several insects (Agblor et al. 1994; Agusti et al. 2000; Cherqui et al. 2000; Cohen et al. 1998; Doostdar et al. 1997; Frati et al. 2006; Laurema et al. 1961; Ma et al. 1990; Shen et al. 1995), the genes encoding polygalacturonases have been characterized from only two insects, the mustard beetle *Phaedon cochleariae* (Coleoptera: Chrysomelidae) (Girard et al. 1999) and the rice weevil *Sitophilus oryzae* (Coleoptera: Curculionidae) (Shen et al. 2003; Shen et al. 1995). Both of these beetles feed on living plants, but neither feeds by the piercing-sucking mechanism found in Heteroptera.

The tarnished plant bug, *Lygus lineolaris* (Palisot de Beauvois) (Hemiptera: Miridae), infests many crops in the eastern United States and Canada and often requires control. *Lygus* spp. damage cotton, alfalfa, seeds, fruits and vegetable crops by inserting stylets, needle-like mouthparts, into plant tissues and injecting saliva that causes tissue necrosis, abscission, and deformation (Strong 1970). *Lygus* spp. salivary enzymes contain polygalacturonases that hydrolyze pectic polysaccharides in plant cell walls (Agblor et al. 1994; Strong et al. 1968). More than one polygalacturonase is present in *Lygus* spp. saliva, and are the primary cause of plant tissue damage (Shackel et al. 2005). Other bugs in the family Miridae also produce polygalacturonases, primarily in salivary glands; interestingly, polygalacturonases have not been detected in other phytophagous heteropteran families (Frati et al. 2006; Laurema et al. 1961).

Molecular genetic characterization of polygalacturonases from agricultural pests, especially those with piercing-sucking feeding mechanisms, will be helpful in formulating and selecting pest resistant crops. Many plants produce polygalacturonase-inhibiting proteins that are being studied for potential use, either through plant selection or genetic manipulation, as defense against fungal and insect damage (D'Ovidio et al. 2004; Howell et al. 2005; Powell et al. 2000; Shackel et al. 2005). Also, pectin-degrading enzymes can be used for food and fiber processing applications (Agblor et al. 1994; Jayani et al. 2005; Shackel et al. 2005; Solbak et al. 2005). Identification of new genes encoding polygalacturonases from plant-feeding mirids will promote progress in agricultural science and technology.

While analyzing and cataloguing a set of cDNA sequences from tarnished plant bug male nymphs (Allen 2007), three sequences homologous to polygalacturonases were found. These sequences indicated three distinct polygalacturonase-encoding genes that were more similar to fungal polygalacturonase genes than polygalacturonases associated with beetles. The presence and dissimilarity of these genes suggested the existence of a broad family of polygalacturonases in phytophagous mirid bugs. All three genes were amplified in their entirety after identification of both ends by rapid amplification of cDNA ends (RACE). Several copies of the genes were amplified using both standard and proofreading polymerase. PCR using specific primers and genomic DNA identified an intron in one of the genes. Stage-specific expression of the three genes was examined by seminquantitative RT-PCR. The characteristics of these genes were compared with insect and fungal counterparts.

## Material and Methods

### Lygus lineolaris

A laboratory colony of *L. lineolaris* was established from specimens supplied by Dr. Eric Villavaso at the USDA ARS Facility in Starkville, MS, and maintained under standard conditions (16:8 L:D at 24°C and 20°C respectively, 70% RH) in Percival (www.percival-scientific.com) incubators at the USDA Stoneville Research Quarantine Facility essentially as described (Allen 2007; Cohen 2000).

### Nucleic acid (RNA and DNA) extraction

Total RNA was extracted from whole live specimens using the Ambion (www.ambion.com) Multi-Enzymatic Liquefaction of Tissue (MELT) total nucleic acid isolation system according to instructions supplied by the manufacturer. Samples were prepared using a DNase digestion step. Polyadenylated RNA was prepared from total RNA samples using the Ambion Poly(A) Purist™ MAG magnetic poly(A) RNA purification kit according to manufacturer instructions. All samples were measured spectrophotometrically to determine concentration and purity. Genomic DNA was extracted using the Promega (www.promega.com) Wizard® SV Genomic DNA Purification System according to manufacturer instructions for preparation of mouse tail and tissue lysates. Samples were crushed in digestion solution containing proteinase K and incubated overnight, then centrifuged and separated from undigested materials prior to column application. Nuclease-free water was heated to 65°C prior to elution for improved yield. Samples were measured spectrophotometrically; if the sample gave low yield or was contaminated with protein (260/230 reading <1.0), the sample was cleaned and concentrated using Zymo (www.zymoresearch.com) DNA Clean & Concentrator-5 column.

### Library construction

Total RNA was extracted (described above) from fifth instar nymph males, which were selected by anesthetizing with CO_2_ and examining the ventral posterior for the absence of a developing ovipositor. Individual males (whole insects) were used for extractions, and total RNA yield varied from 6.3 µg to 24.2 µg per specimen. Six samples with yields >15 µg were combined and used to purify poly(A) RNA. The poly(A) RNA (2.4 µg) was used to prepare a cDNA library as described (Allen 2007) using the Invitrogen (www.invitrogen.com) Superscript™ plasmid system with Gateway® Technology for cDNA synthesis and cloning, according to instructions provided, except that quantification of concentration of size fractions was calculated spectrophotometrically. A fraction containing transcripts of about 1 kb in length was ligated into the pSPORT1 vector, and then transformed into One Shot®] OmniMAX™ 2 T1 phage-resistant cells.

### RACE and full-length cloning

Ends of the cDNA encoding putative polygalacturonases were obtained using the Invitrogen GeneRacer™ kit according to instructions provided by the manufacturer. Total RNA samples were treated with both calf intestinal phosphatase and tobacco acid pyrophosphatase to facilitate 5′ cloning. All PCR primers were designed using Primer3 (http://frodo.wi.mit.edu/cgi-bin/primer3/primer3_www.cgi) (Rozen et al. 2000), and PCR was performed using Applied Biosystems AmpliTaq Gold® polymerase. RACE products were gel purified and cloned into Invitrogen pCR 4-TOPO plasmids, then transformed into One Shot® TOP10 chemically competent cells. After sequences were manually assembled into full-length genes, first strand cDNA was reamplified with Applied Biosystems GeneAmp® high fidelity polymerase using primers designed to amplify the entire open reading frame. To identify possible introns, additional primers were designed and similarly used to amplify genomic DNA. The amplicons were cloned and sequenced, and the resulting sequences were used to reconcile the gene sequences assembled from ESTs and RACE products.

### Sequencing and Analysis

Clones were sequenced on an Applied Biosystems ABI3730 at the USDA Genomics Center in Stoneville, MS and compared with nucleotide and translated proteins published in GenBank using the NCBI BLAST analysis programs blastn and tblastx (Altschul et al. 1997). Open reading frames were identified and translated and protein characteristics identified using DNASTAR Lasergene software. Multiple alignments were performed using MEGA 3.0 software (Kumar et al. 2004), or submitted to the CLUSTAL W (version 1.82) online service (http://www.ebi.ac.uk/clustalw) (Higgins et al. 1994). Phylogenetic comparisons were made using the neighbor joining method and reconstructed with 1000 replicate bootstrap analysis. Signal peptides were identified using the SignalP 3.0 online server (Bendtsen et al. 2004).

### Semiquantitative RT-PCR

Total RNA samples were diluted to a concentration of 100 ng/µl and 500 ng was used in reverse transcription reactions using Ambion M-MLV reverse transcriptase, RNase inhibitor, and Oligo(dT) primers following manufacturer instructions. The number of cycles required to produce visible bands but avoid saturation was determined empirically to analyze developmental stages of insects. Typically, 26 cycles were used for *L. lineolaris* polygalacturonase. Typical cycling conditions were: 2 min. 94°C initial denaturation step followed by repeated cycles of 94°C for 30s, 58°C for 20°, 68°C for 70s, and final extension for 10 min at 68°C. Primers used to amplify full-length genes were also used for expression evaluations. Experiments with insect stages were performed twice, and because total RNA from adult specimens represented individuals, six additional male and six additional female adults were used in a follow-up experiment. Expression controls were chosen from several sequences that were expected to be expressed constitutively (at all stages of development) and evaluated while the semiquantitative cycling parameters were empirically determined. *L. lineolaris* cDNA library sequences homologous to cytoplasmic actin (DQ386914), ribosomal S2 (DY470915) and 16S (DY524581) were expressed in all samples. Control samples required 22 PCR cycles. Relative expression levels were estimated and normalized to the controls from gel band density measurements calculated using TotaLab TL100 gel analysis software.

**Figure 1.**
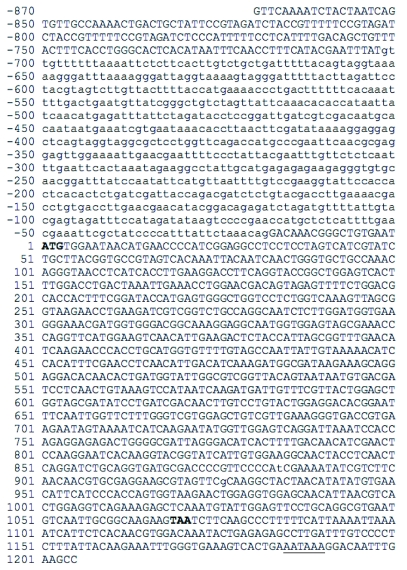
Genomic sequence of *Lygus lineolaris* polygalacturonase1 (PG1). Lowercase letters denote intron in the 5′ untranslated region. Start and stop codons are indicated in bold. Putative polyadenylation signal is underscored. GenBank accession number EU136628.

**Table 1.**
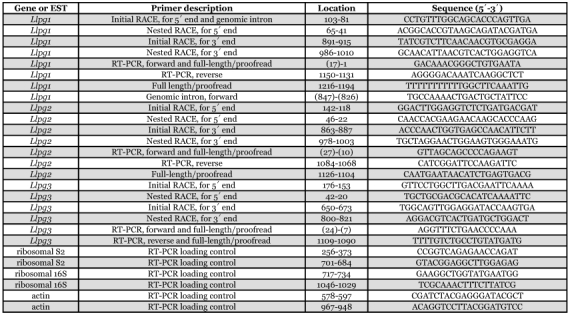
Primers used to amplify polygalacturonase and loading control (putative identifications based on homology) genes complementary and genomic from *Lygus lineolaris* DNA.

## Results

Expressed sequenced tags from a cDNA library (Allen 2007) were preliminarily identified as polygalacturonase encoding genes based on similarity to known fungal sequences. The three library sequences were compared to one another and found to be highly divergent at the nucleotide level, except for a 65 nt portion of *Llpg2* and *Llpg3*, with 53/65 (81%) identities and no gaps. The corresponding translation of these nucleotides (respectively, PG2/PG3 for nonidentities) is K-P-G-T-T-V / I-E / V-F-A / R-G-R-I / V-T-F-G-Y-K-E-W-K / R-G-P. All three expressed sequenced tags differed at the nucleotide level from all other sequences in NCBI/GenBank. The low sequence similarity between the three expressed sequenced tags and their moderate translated amino acid similarities to several fungus and both insect polygalacturonases indicated that these were probably unique products, so the genes were cloned in their entirety using RACE, followed by amplification of complete coding sequences from cDNA. Specific primers used for all three genes are shown in Table 1. When the specific primers were used to try to amplify genomic sequences, multiple bands were obtained, suggesting the existence of either introns, multiple isoforms, or pseudogenes within the genome. For *Llpg1* the genomic amplicons were longer than the cDNA sequence, so specific primers (Table 1) were used to amplify a 688 bp intron in the 5′ untranslated region. The deduced genomic sequence for *Llpg1* is shown in Figure 1. The three cDNA sequences were submitted to the NCBI/GenBank database as CoreNucleotides under accession numbers DQ399525, DQ399526, and DQ399527.

**Figure 2.**
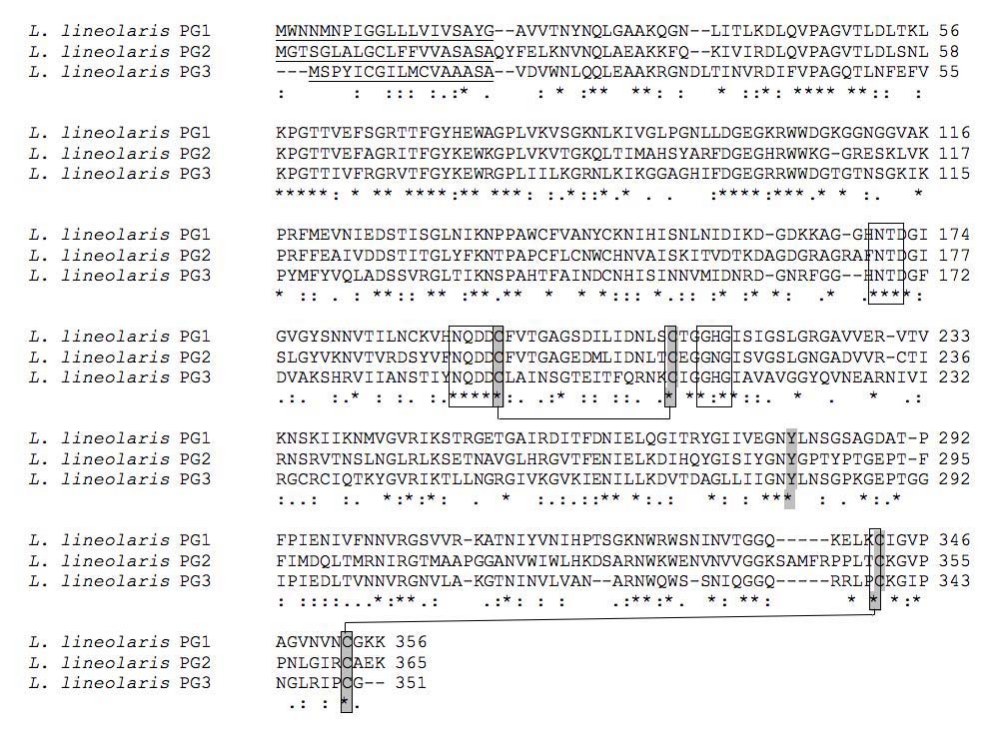
Multiple sequence alignments of the deduced amino acid sequences. (A) *L. lineolaris* polygalacturonase (PG) cDNAs. ^*^ marks identities, : marks functionally conservative substitutions, . marks majority (2 of 3 either identical or functionally conserved). Predicted signal peptides are underscored. Conserved functional motifs are boxed, cysteines are shaded and lines connect putative disulfide bridges. Strictly conserved Tyr281 is shaded. (B) Multiple alignment of fungal and insect PGs.

The three cDNA's encode putative polygalacturonases of 356, 365, and 351 amino acids for *Llpg1, Llpg2* and *Llpg3,* respectively (Figure 2A). The LlPG1 enzyme was predicted to have a 20 amino acid signal peptide (probability 0.892, cleavage site probability 0.810) (Bendtsen et al. 2004) with the molecular weight of the mature enzyme predicted to be 35.8 kD. The LlPG2 protein is also predicted to have a 20 amino acid signal peptide (probability 1.0, cleavage site probability 0.948) and a molecular weight of 38.1 kD for the mature peptide. LlPG3 is predicted to have a 17 amino acid signal peptide (probability 0.989, cleavage site probability 0.906) leading to a predicted molecular weight of 36.5 kD for the mature peptide. The amino acid sequences of the three putative enzymes as would be expected are also highly divergent with LlPG1 having 49% identity to LlPG2 and 44% identity LlPG3, while LlPG2 shares 39% identity with LlPG3 (Figure 2A).

**continued:**
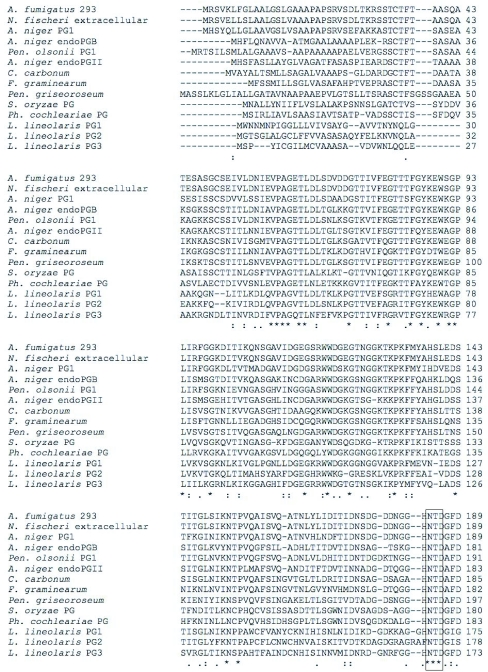

**continued:**
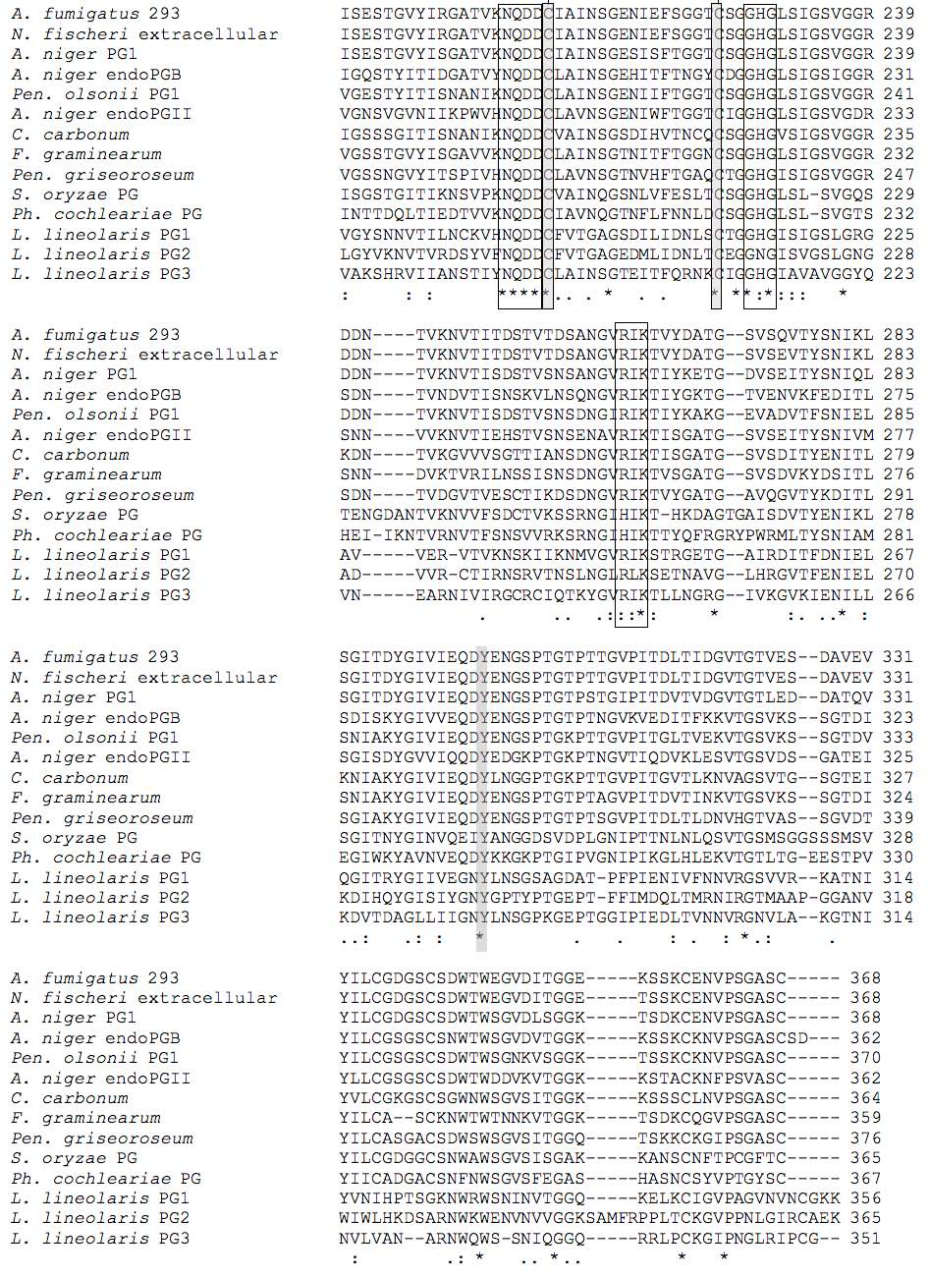

Despite the low degree of sequence identity found among the putative *L. lineolaris* polygalacturonases, the three enzymes contain the strictly conserved amino acid residues found across all fungal and bacterial polygalacturonases isolated to date (Kester et al. 1996). The three strictly conserved aspartic acid residues involved in catalysis (Armand et al. 2000; Pickersgill et al. 1998; van Santen et al. 1999), the consecutive aspartic acid residues (Asp193 and Asp194, LlPG1 numbering used throughout) along with Asp 172, are conserved in all three putative *L. lineolaris* polygalacturonases (Figure 2B). The putative enzymes also contain the strictly conserved residues Arg246, Lys248, and Tyr281 (Figure 2B). LlPG1 and LlPG3 possess the strictly conserved histidine residue (His215); however LlPG2 substitutes an asparagine residue at this position (Figure 2A). The impact of this substitution on LlPG2 is unclear, but may lead to an inefficient catalyst as the His215 residue has been implicated in substrate binding of *A. niger* endo-PG II (van Santen et al. 1999). The *L. lineolaris* polygalacturonases contain two potential disulfide bridges, a departure from the four disulfide bridges found in fungal endo-polygalacturonases. One potential disulfide bridge involves Cys195 and Cys 211, very near the active site (Figure 2A). This disulfide bridge connects two adjacent β-helical turns in *A. niger* and *A. aculeatus* polygalacturonase crystal structures (Cho et al. 2001; van Santen et al. 1999). The second potential disulfide bridge is found at the C-terminus of the three enzymes, Cys342 and Cys353 (Figure 2A), and is expected to block access to the core of the β-helix structure common to family 28 polygalacturonases (Cho et al. 2001; Markovic et al. 2001; van Santen et al. 1999).

**Table 2.**
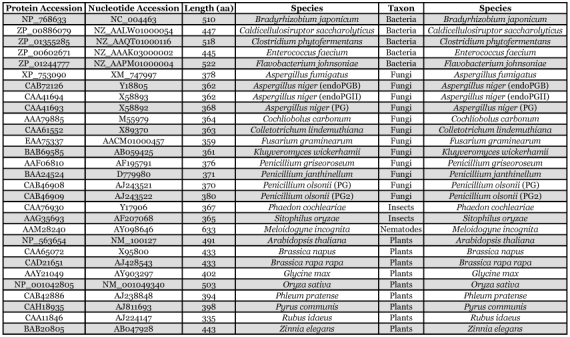
Proteins used for multiple alignment and phylogenetic reconstruction, species names and the broad taxa to which they belong.

**Figure 3.**
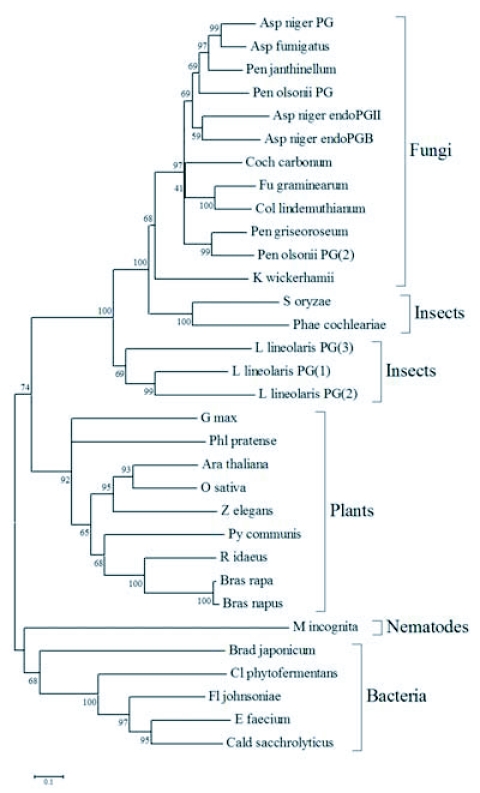
Phylogenetic tree of some polygalacturonase proteins constructed by neighbor-joining (NJ) criteria. Bootstrap support values are shown at nodes, scale is 0.1 amino acid substitution per site. GenBank accession numbers and full species names for each sequence can be found in Table 2.

A polygalacturonase phylogenetic tree was constructed to facilitate comparison of polygalacturonases from *L. lineolaris*, two other insects, and other taxa. The fungal, plant, and bacterial polygalacturonase sequences used were those identified by tblastx to be the most similar to insect polygalacturonases. The single nematode polygalacturonase described to date, that from *Meloidogyne incognita*, was also included. Species used for analysis are shown in Table 2, and the tree is presented in Figure 3. While the insect polygalacturonases were clearly most closely related to fungal polygalacturonases, the insect polygalacturonases were distinctly separated from the fungi, albeit in two groups. The two polygalacturonases identified from beetles, *S. oryzae* and *P. cochleariae,* formed a group phylogenetically separate from the three Lygus polygalacturonases (Figure 3).

**Figure 4:**
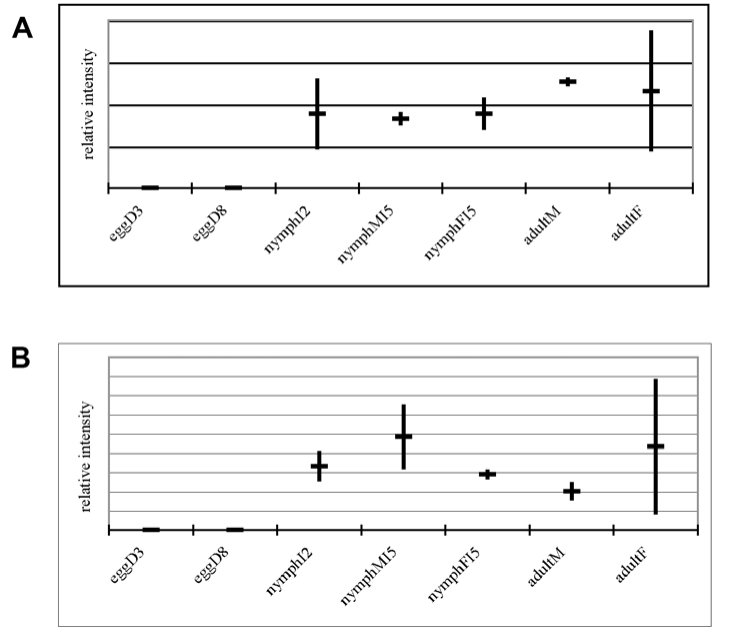
Expression profiles of polygalacturonase genes of *Lygus lineolaris.* (A-C) Relative semiquantitative RT-PCR values, as determined by gel density analysis and normalized to cytoplasmic actin, 16S ribosomal, and S2 ribosomal standards. Vertical bars connect the two expression levels in separate experimental replicates, horizontal crossbars indicate the average. EggD3: 3 day-old eggs, eggD8: 8 day-old eggs, nymphI2: second instar (small) nymphs, nymphMI5: fifth instar male nymph, nymphFI5: fifth instar female nymph, adultM: male adult, adultF: female adult. (A) *Llpg1*, (B) *Llpg2*, (C) *Llpg3*. (D) Semiquantitative RT-PCR gels of additional adult specimens. F: female, M: male, O: no template, PG1: *Llpg1*, PG2: *Llpg2,* PGs: *Llpg3*, S2: S2 ribosomal standard.

To gain functional insight into the of *Llpg* genes, transcriptional profiling was performed at various developmental stages. RT-PCR was carried out using RNA reverse transcribed from eggs at an early and late stage of development, approximately day 3 and day 8 respectively; from small, whole second instar nymphs; from fifth instar, pre-adult nymphs that could be segregated by sex; and from adult males and adult females. The sample sizes were based on calculated biomass of 10 mg, therefore a total RNA extract was prepared from 150 eggs, 12 small nymphs, 2 whole preadult nymphs, and 1 whole adult. Replicate samples were prepared, and a series of semiquantitative amplifications confirmed that the *Llpgs* were not expressed in eggs but were expressed in all feeding stages (Figure 4 A-C). Expression appeared highly variable both within and between stages and sexes. Because the adult samples represented so few individuals, additional samples were prepared from whole adults and semiquantitative PCR was repeated, using the same cycling program (Figure 4 D). Expression controls, *L. lineolaris* sequences homologous to cytoplasmic actin, ribosomal S2 and 16S, were expressed at all stages of development (primers shown in Table 1) at similar intensity. Variability of *Llpg* expression in adult specimens, and S2, is illustrated in Figure 4 D.

## Discussion

Three cDNAs encoding three unique putative polygalacturonases were isolated and completely sequenced. The *L. lineolaris* genes encode proteins that are similar to other insect polygalacturonases in both sequence, predicted signal peptide and predicted molecular weight (Girard et al. 1999; Shen et al. 2003; Shen et al. 1995). The deduced protein products of the *L. lineolaris* genes, as well as the previously isolated insect polygalacturonases, are most closely related to fungal polygalacturonases (Figure 3). Shen et al. (2003) hypothesized that the *S. oryzae* polygalacturonase genes may have been horizontally transferred from a fungal species.

The *Llpg* translation products contain many characteristic features of polygalacturonases including four strictly conserved segments and an invariantly conserved tyrosine residue (Markovic et al. 2001). However, some semi- or nonconserved characteristics may prove responsible for enzymatic differences when further studied. One exception to the consensus was an asparagine residue substituted for the conserved His215 in LlPG2. This histidine is strictly conserved in fungal polygalacturonases and when mutated in *A. niger* endo-PG II led to an enzyme with less than 5% of the wild-type activity (van Santern et al. 1999). The histidine residue is thought to play a role in substrate binding (van Santern et al. 1999); however the impact of this substitution on potential LlPG2 activity is unclear and will require more detailed studies of the isolated enzyme.

The *L. lineolaris* polygalacturonases potentially contain only two of the four disulfide bridges (Figure 2) found in polygalacturonases from coleopterans and fungi. This is not unexpected as structure and function are likely to differ between beetle, mirid and fungal polygalacturonases due to the environments found in the insect guts and fungal environs versus the *L. lineolaris* polygalacturonases that are secreted into saliva that is injected into a plant for extraoral digestion of the cell walls. The *L. lineolaris* enzymes are unique in that the disulfide bridge found near the N-terminus of all previously isolated polygalacturonases does not exist in the putative LlPGs. This disulfide bridge, in combination with other structural features, serves as a shield to the inner core of the β-helix structure expected to be common among all family 28 hydrolases (Cho et al. 2001). How the *L. lineolaris* enzymes accomplish the shielding of the β-helix core, and what impact it may have on enzyme activity and/or stability will require more studies. The enzymes also do not have what may be referred to as the 3rd disulfide bridge that provides structural stability in the *A. nicer* endo-polygalacturonase II enzyme (van Santen et al. 1999), however this disulfide bridge is not strictly conserved across other species. The putative *L. lineolaris* polygalacturonases do contain the disulfide bridges near the active site and the C-terminal end. The C-terminal disulfide bridge, which also acts as a shield for the β-helix core, is present in the *L. lineolaris* enzymes and is similar to the bridges found in plant, insect, and fungal polygalacturonases, but not bacterial polygalacturonases. This is an important distinction as this supports the assumption that the genes isolated here are indeed of *L. lineolaris* origin.

**continued:**
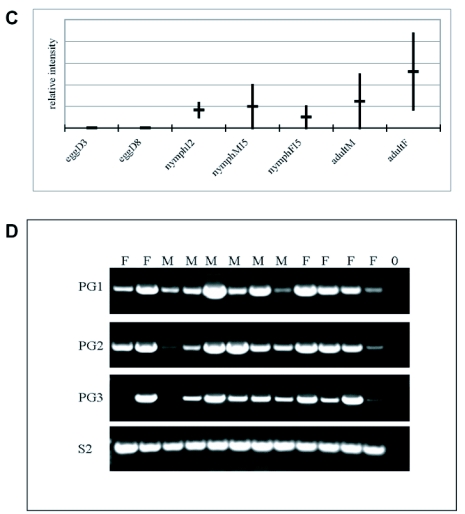

When a similar polygalacturonase gene was cloned from the rice weevil, *S. oryzae*, the possibility that it was of endosymbiont rather than insect origin was raised. This question was carefully examined, and the effort led to the conclusion that the sequence was a single copy polygalacturonase gene in the insect genome (Shen et al. 2003). However, because the *S. oryzae* polygalacturonase gene from was shown to be more similar to fungal polygalacturonase genes than to any known insect genes, the authors speculated that the gene was transferred horizontally from a fungus to an early coleopteran (Shen et al. 2003). The *P. cochleariae* polygalacturonase was also concluded to have originated from the insect genome, based on screening of the microbial fauna of the insect at several insect life stages, and the presence of 3′ poly(A) tails (Girard et al. 1999). The *L. lineolaris* cDNA sequences from which the polygalacturonase genes were identified, although admittedly few, contained no other sequences that would indicate the presence of endosymbiotic organisms (Allen 2007). Despite this genus being the focus of more study than most of the remainder of the family Miridae, and despite the documented presence of endosymbionts in many heteropteran and homopteran relatives, no endosymbionts have been found in a *Lygus* species (Kikuchi et al. 2003). Additionally, documented pathogen transmission is rare in *Lygus* (Wheeler 2001). The three polygalacturonase genes described here are more closely related to one another and to the other insect polygalacturonases than to the most similar fungal polygalacturonases when analyzed phylogenetically (Figure 3 and Table 2). It is almost certain that these three genes are of *L. lineolaris* genomic origin. The evolutionary origin of these genes is an area for more study. As additional insect polygalacturonases are identified and characterized it is likely that one or more distinct insect lineages will emerge.

If the three polygalacturonase sequences identified from *L. lineolaris* are salivary or digestive enzymes, they should be expressed in feeding stages of the insect but not in the egg stage. Because three distinct genes were identified, it is possible that they are differentially expressed based on development, or sex, or some other factor. The *L. lineolaris* cDNA library was derived from fifth instar male nymphs (Allen 2007), warranting a determination of expression from other life stages. In each replicate of the stage expression experiments, a zero expression reading was present in at least one life stage for *Llpg3*. This, and the overall variability in expression indicated in Figure 4, suggests that all three genes are differentially expressed in all feeding stages, and are regulated by factors other than development or sex. Salivary protein profiles from *L. hesperus* varied depending on diet and starvation status (Habibi et al. 2001). Enzymes extracted from *L. hesperus* and *L. lineolaris* vary in specific activity between species and tissue (Agusti et al. 2000) and depending on starch source (Agblor et al. 1994). The range of digestive enzymes produced by *L. hesperus* and *L. lineolaris* is indicative of the polyphagous nature of these bugs; they not only attack plants, but are facultative predators as well (Agusti et al. 2000; Wheeler 2001). All of the specimens in the expression studies here were laboratory reared on artificial diet. Future expression assays using a variety of diets may pinpoint host cues that regulate expression of specific polygalacturonase genes. Furthermore, libraries derived from Lygus salivary glands rather than whole insect specimens should provide a more complete set of polygalacturonase and other enzymes involved in extraoral feeding. We have begun preparation of these resources.

Purified fungal genes for carbohydrate-degrading enzymes have been useful for analyses of plant cell walls (Bauer et al. 2006). Degradation of pectins is important in food technology and waste management; the *Lygus* spp. enzymes may be industrially useful (Agblor et al. 1994). The specificity and activity of the *L. lineolaris* enzymes, and those from other insects, may prove useful in a number of applications. Perhaps most importantly, the interaction between *Lygus* spp. polygalacturonases and plant defensive proteins such as polygalacturonase-inhibiting proteins may lead to novel crop protection strategies.

In conclusion, the first three polygalacturonases from *L. lineolaris* have been identified. Additional gene identifications can be expected from this and other *Lygus* species as well as from other phytophagous members of the family Miridae. The genes are transcribed in all feeding stages and appear to be highly inducible. The stimuli that drive expression of individual or multiple polygalacturonases can now be tested, and the specific characteristics and functions of the individual or combined forms will provide a basis for understanding, combating, and possibly exploiting this pest.

## Abbreviations

Llpg -: *Lygus lineolaris* polygalacturonase
RACE -: rapid amplification of cDNA ends
RT-PCR -: reverse transcription polymerase chain reaction
